# Understanding normal cardiac morphogenesis and its disruptions: a journey through pathways

**DOI:** 10.3389/fgene.2026.1753998

**Published:** 2026-07-15

**Authors:** Aline L. Saliba, Jorge Afiune, Aline Pic-Taylor, Silviene F. Oliveira, Juliana F. Mazzeu

**Affiliations:** 1 Programa de Pós-graduação em Ciências Médicas, Universidade de Brasília, Brasília, Brazil; 2 Hospital da Criança de Brasília José de Alencar, Brasília, Brazil; 3 Instituto de Cardiologia e Transplantes do Distrito Federal, Brasília, Brazil; 4 Departamento de Genética e Morfologia, Instituto de Ciências Biológicas, Universidade de Brasília, Brasília, Brazil; 5 Instituto Nacional de Doenças Raras (InRaras), Universidade de Brasília, Brasília, Brazil; 6 Faculdade de Medicina, Universidade de Brasília, Brasília, Brazil

**Keywords:** cardiogenesis, congenital heart disease, developmental biology, genetic variants, signalling pathways, transcription factors

## Abstract

Congenital heart diseases (CHDs) encompass a broad spectrum of structural anomalies with substantial clinical and genetic heterogeneity. They are the most common birth defects in humans, and a leading cause of paediatric morbidity and mortality. Yet, its genetic substrate remains difficult to interpret at the bedside: despite advances in cytogenetics and next-generation sequencing, a definitive or candidate genetic cause is identified in fewer than half of cases, and even when a variant is recovered, mapping it onto the developmental program that produces a specific malformation is rarely straightforward for the practising clinician. This narrative review revisits normal cardiogenesis as a single, coordinated developmental program, integrating embryological events with progenitor populations, transcription factor networks, and signalling pathways. We then highlight how perturbation of these developmental modules may result in syndromic and non-syndromic CHD. By aligning embryological events with their regulatory logic, the review offers a developmental framework intended to help clinicians situate molecular findings within the biology of heart formation, sharpen genotype–phenotype interpretation, support more precise diagnostic and prognostic reasoning, and inform emerging regenerative strategies for the malformed and injured heart.

## Introduction

Congenital heart disease (CHDs) consists of a heterogeneous group of malformations of the heart and great arteries and represents the most common birth defect in humans, with an estimated prevalence of 4-12 in 1000 (0,4 – 1,2%) live births (Ison et al., 2022; Pierpont et al., 2018; Suluba et al., 2020) although its true incidence cannot be measured accurately without the rates of spontaneous and planned pregnancy terminations (Liu et al., 2023) and late diagnosis (Pierpont et al., 2018). The incidence may thatincrease by up to 3% when minor lesions or defects with spontaneously close are included (Van Der Bom et al., 2011; Zimmerman et al., 2020).

About one-third of the children born with CHD have severe defects that demand interventions in the first years of life (Zaidi and Brueckner, 2018), mainly with a palliative approach. Even those with minor anomalies will most likely have sequelae of either the native disease or its surgical repair (Stout et al., 2019), although the cumulative impact of exposure to CHD on mortality is still unknown (Nelson et al., 2022). Patients with corrected or palliated CHD are at more risk of developing arrhythmias and myocardial dysfunction (Zaidi and Brueckner, 2018), among other perioperative or later complications. However, the potentially most considerable effect on the quality of life and high costs to society are from associated neurodevelopmental disabilities (Zaidi and Brueckner, 2018; du Plessis and d’Udekem, 2019) in which perioperative factors such as anaesthesia or cardiopulmonary bypass have been primarily disproven to play a significant role, accounting for less than 5% of the disruption in early neurodevelopment. Instead, a more complex set of innate factors appears to be responsible (du Plessis and d’Udekem, 2019; Nattel et al., 2017).

Notwithstanding, steady advancements in surgical procedures, hemodynamic management and diagnostics have improved the survival of patients, leading to an evident change in the epidemiology of CHDs, with adults outnumbering children with this condition (Khan and Gurvitz, 2018). Further amelioration in survival demands a better understanding of patient-specific risk factors and uncovering the etiology of CHDs becomes increasingly significant for assessing recurrence risk in the family of the patients, evaluating extracardiac involvement, especially neurodevelopmental and providing a more accurate prognosis (Pierpont et al., 2018; Russell et al., 2018). Genetic syndromes and nonsyndromic genetic variations significantly affect long-term survival after surgical repair or palliation (Russell et al., 2018).

Patients with CHDs are a very heterogeneous population, which can be a significant challenge for the epidemiological study regarding prevalence, prevention, prognosis, and incidence in survivors' offspring (Khan and Gurvitz, 2018).

Aneuploidies and large copy number variations may account for 18% of CHDs (Pierpont et al., 2018; Zaidi and Brueckner, 2018; Russell et al., 2018). Extensive studies with Next-Generation Sequencing (NGS) suggest that 8%-20% of cases are attributable to *de novo* autosomal dominant variants, and 2% are related to inherited autosomal recessive variants (Pierpont et al., 2018; Morton et al., 2022). However, models of monogenic inheritance raise essential questions regarding genotypic and phenotypic variance (Suluba et al., 2020).

Collectively, these approaches identify damaging coding variants in definitive and candidate genes for CHD in 45% of patients (Morton et al., 2022). Environmental exposures account for 2% of CHD instances; the unexplained cases are presumed oligogenic or multifactorial (Pierpont et al., 2018).

Since cardiogenesis is a highly complex and very early developmental process, it is unsurprising that CHDs are the most common congenital anomalies since it is particularly susceptible to changes in gene dosage among countless pathways. Variants in more than 400 genes are suggested to cause human cardiac anomalies, and genotype-phenotype correlations suggest that specific morphogenetic mechanisms induced and modulated by gene networks and specific cellular pathways may result in specific phenotypes (Calcagni et al., 2021). Therefore, understanding normal heart formation may provide new insights into the etiology of CHD, and data about genes associated with anatomical or physiological variations are informative about subtler influences on the embryonic heart.

In this narrative review, we revisit cardiogenesis as a coordinated developmental program in which progenitor specification, morphogenetic events, and regulatory networks interact to shape the forming heart, and consider how disturbances in these processes may lead to congenital heart diseases. Since the multitude of genes would require an exhaustive treatment, beyond the scope of any single review, we restricted our discussion to transcription factors and signalling pathways that satisfy at least two of the following criteria: (i) experimentally validated roles in human cardiogenesis, supported by hiPSC, organoid, or human genetic studies; (ii) recurrent identification in CHD patient cohorts, with established genotype–phenotype correlations; and (iii) the capacity to integrate multiple developmental modules. The main genes discussed in this review are summarized in Table 1.

**TABLE 1:** Genes and signalling components discussed in this review are listed alphabetically. For each entry, we indicate the pathway or family, role in cardiogenesis, the model system(s) in which the cited evidence was generated, the associated congenital heart disease (CHD) phenotype where one has been described in humans, and the corresponding references in the bibliography of this manuscript.

Gene	Pathway or family	Role in cardiogenesis	Model system(s)	Associated CHD phenotype/ Syndrome	Referencess
*BMP2*	TGF-β/ BMP superfamily	Inhibited by Notch-activated HEY1/HEY2 in chamber myocardium; expression restricted to the AVC.	Mouse	–	Li et al. (2021)
*BMP4*	TGF-β/ BMP superfamily	Activates SMAD1/5-mediated transcriptional responses; with Wnt and Nodal signalling, promotes emergence of early cardiac mesoderm and precedes EOMES activation. Also enriched in human SAN-related gene set.	Mouse + human	–	Tsaytler et al. (2023), Park and Fishman (2026), Kapoor et al. (2013)
*BMP10*	TGF-β/ BMP superfamily	Atrial-program marker driven by TBX5.	Mouse	–	Sweat et al. (2023)
*CERL2*	Nodal antagonist	Right-side-biased expression at the LRO antagonising NODAL; mutually antagonistic feedback with WNT3.	Mouse	–	Djenoune et al. (2022), Dykes (2014), Little and Norris (2021)
*DAND5*	Nodal antagonist	Asymmetrically expressed on the right side of the LRO; competes with NODAL to prevent inappropriate left-sided signalling.	Mouse + human	Heterotaxy (autosomal-recessive variants)	Sempou and Khokha (2019), Wells et al. (2022)
*EOMES (Eomesodermin)*	T-box transcription factor	Earliest committed cardiac precursor marker; activates MESP1 and enables progenitors to emerge from the primitive streak.	Mouse	–	Tsaytler et al. (2023), Bondue and Blanpain (2010), Bruneau (2013)
*FGF10*	FGF signalling ligand	SHF signalling molecule downstream of the ISL1/FOXH1 axis; required for SHF development.	Mouse	–	Wang et al. (2016), Qyang et al. (2007), Ren et al. (2021)
*FOXC1, FOXC2*	Forkhead transcription factors	MESP1 targets in the cardiovascular TF cascade; FOXC2 is also a direct TBX1 transcriptional target.	Mouse	–	Bondue and Blanpain (2010), Bondue et al. (2008), Nomaru et al. (2021)
*FOXH1*	Forkhead transcription factor	MESP1 target; cooperates with ISL1 in the SHF regulatory network; partners with Smad-dependent components in the L-R NODAL cascade upstream of *PITX2.*	Mouse	–	Bondue and Blanpain (2010), Bondue et al. (2008), Desgrange et al. (2018), Kato (2011)
*GATA4*	GATA zinc-finger TF	FHF marker; cooperates genome-wide with TBX5 for chamber specification (LV, septum); acts sequentially with NKX2-5 and TBX5; regulates cardiomyocyte proliferation/survival, vascular integrity and ECM. Cooperates with TBX5/TBX20/NKX2-5 in fast-conducting gene programmes.	Mouse + human iPSC	Septal, valvular, outflow-tract defects (broad CHD spectrum)	Miyamoto et al. (2021a), Canac et al. (2022), Ang et al. (2016), Gonzalez-Teran et al. (2022), Bondue and Blanpain (2010), Bondue et al. (2008), Heuvelmans et al. (2025), Park and Fishman (2026)
*GDF1*	TGF-β family co-ligand	Forms a heterodimer with NODAL to propagate the left-sided signal into the LPM.	Mouse + human	Heterotaxy (autosomal-recessive variants)	Wells et al. (2022)
*GJA1 (CX43)*	Connexin/ gap junction	Repressed by TBX18; loss of repression enables biological-pacemaker conversion of ventricular cardiomyocytes.	Mouse	–	Park and Fishman (2026), Kapoor et al. (2013)
*GJA5 (CX40)*	Connexin/ gap junction	Atrial marker driven by TBX5; repressed by HEY2 in ventricular myocardium; marks early chamber myocardium distinct from TBX3+ CCS.	Mouse	–	Sweat et al. (2023), Seya et al. (2021), Park and Fishman (2026), Mohan et al. (2018)
*HAND1*	bHLH transcription factor	FHF marker; also marks a MESP1+ Juxtacardiac Field population. Promoted by IRX4 in the ventricular gene programme.	Mouse	–	Miyamoto et al. (2021a), Canac et al. (2022), Sendra et al. (2022), Tyser et al. (2021), Zhang et al. (2021), Nelson et al. (2016)
*HAND2*	bHLH transcription factor	MESP1 target induced as part of the early cardiovascular TF programme.	Mouse	–	Bondue and Blanpain (2010), Bondue et al. (2008)
*HCN4*	HCN cation channel	FHF marker. Predominant pacemaker channel in the SAN	Mouse + human	–	Liang et al. (2013), Wu et al. (2025), Hennis et al. (2021)
*HEY1, HEY2*	Notch downstream bHLH repressors	Notch-activated; inhibit BMP2 and TBX2 in chamber myocardium to restrict their expression to the AVC. HEY2 is a major determinant of ventricular morphology, repressing atrial-specific genes	Mouse + hiPSC	Tricuspid atresia (mouse).	Seya et al. (2021), Okumura et al. (2024), Li et al. (2021)
*IRX3, IRX4, IRX5*	Iroquois homeobox TFs	Establish regional ventricular identity and electrical maturation.	hiPSC + mouse	–	Canac et al. (2022), Wang et al. (2013), Kim et al. (2012), Nelson et al. (2016)
*ISL1*	LIM-homeobox TF	Definitive SHF marker; partners with the BRG1/BAF60c chromatin complex; supports SHF progenitor maintenance and deployment; contributes to RV, OFT and partly atria;	Mouse + human	–	Liang et al. (2013), Wu et al. (2025), Wang et al. (2016), Ren et al. (2021), Park and Fishman (2026), Kapoor et al. (2013)
*JAG1*	Notch	Notch ligand	Human	Alagille syndrome	Liu et al. (2026), Luxán et al. (2016)
*LEFTY1, LEFTY2*	Nodal antagonists	LEFTY1 acts as a midline barrier preventing NODAL spread to the right side; LEFTY2 (induced by PITX2) restricts the self-enhancing NODAL loop in the LPM in space and time.	Mouse	–	Sempou and Khokha (2019), Desgrange et al. (2018), Little and Norris (2021)
*MAB21L2*	Conserved cell-cycle regulator	Defining transcriptomic signature of the Juxtacardiac Field (JCF); After heart tube formation, MAB21L2+ cells restrict to dorsal mesoderm and source the pro-epicardium, expressing *BNC2, WT1, ITGA8.*	Mouse (single-cell + lineage tracing)	–	Sendra et al. (2022), Tyser et al. (2021)
*MEF2C, MYOCD*	MADS-box TF/ transcriptional coactivator	MESP1 targets and components of the SHF cascade; contribute to cardiomyocyte structural and contractile programmes.	Mouse	–	Bondue and Blanpain (2010), Bondue et al. (2008), Wang et al. (2016), Qyang et al. (2007), Ren et al. (2021)
*MESP1*	bHLH transcription factor	Apex of the cardiac transcriptional hierarchy; orchestrates emergence, migration and early segregation of cardiac progenitors; binds promoters of major cardiac TFs (HAND2, NKX2-5, GATA4, MEF2C, TBX20, FOXH1, FOXC1, FOXC2).	Mouse	–	Bondue and Blanpain (2010), Bruneau (2013), Bondue et al. (2008)
*MYL3, RYR2, TTN*	Sarcomeric/ contractile genes	Downstream of the SHF cascade; cardiomyocyte structure and contraction.	Mouse	–	Wang et al. (2016), Qyang et al. (2007), Ren et al. (2021)
*NKX2-5*	Homeobox TF (NK family)	FHF marker; cardiomyocyte specification; participates in the GATA4–NKX2-5–TBX5 cascade and in fast-conduction gene programmes. Downregulated in MAB21L2+ JCF cells.	Mouse + human iPSC	Septal defects (broader CHD associations)	Canac et al. (2022), Bondue and Blanpain (2010), Bondue et al. (2008), Wang et al. (2016), Qyang et al. (2007), Ren et al. (2021), Park and Fishman (2026)
*NODAL*	TGF-β/ BMP superfamily morphogen	Asymmetrically expressed on the left side of the LRO to establish the L-R programme; propagates to LPM via heterodimer with GDF1; restricted in space/time by LEFTY1, LEFTY2, CERL2, DAND5.	Mouse + multiple vertebrates	Heterotaxy spectrum (autosomal-dominant Nodal/TGF-β variants)	Djenoune et al. (2022), Dykes (2014), Desgrange et al. (2018), Wells et al. (2022), Little and Norris (2021)
*NOTCH1*	Notch receptor	Multi-stage role: cardiac mesoderm patterning, heart-field allocation, endocardial cushion EMT, valve formation, OFT development, ventricular trabeculation/compaction. HiPSC: disruption shifts cells toward epicardial/SHF fates and against FHF.	hiPSC + mouse	Hypoplastic Left Heart Syndrome; left-sided malformations; ventricular septal defects; semilunar valve malformations	Ye et al. (2023), Miyamoto et al. (2021b), High et al. (2009), Li et al. (2021)
*NPPA (ANF)*	Natriuretic peptide gene	Atrial-specific marker driven by TBX5; repressed by HEY2 in ventricular cardiomyocytes; suppressed by IRX4 in ventricular gene programme.	Mouse	–	Sweat et al. (2023), Seya et al. (2021), Nelson et al. (2016)
*NR2F2 (COUP-TFII)*	Nuclear receptor	Most prominent determinant of atrial identity in vertebrates; binds loci near cardiac morphogenesis genes including *TBX5, HEY2, IRX4.*	Mouse	–	Martin and Waxman (2021), Wang et al. (2013)
*PITX2*	Paired-like homeodomain TF	Primary downstream effector of left-sidedness;	Mouse	Heterotaxy	Sempou and Khokha (2019), Desgrange et al. (2018), Little and Norris (2021), Kato (2011)
*SCN5A*	Voltage-gated sodium channel α-subunit	Endpoint of the IRX3/IRX5 → GATA4–NKX2-5–TBX5 cascade; links ventricular patterning to chamber-specific electrical properties.	Mouse + human iPSC	Brugada syndrome	Canac et al. (2022), Park and Fishman (2026)
*SEMA3D, SCX*	Pro-epicardial markers	*SEMA3D*+ and *SCX*+ pro-epicardial populations contribute to sinus venosus and coronary vascular lineages, respectively.	Mouse	–	Feulner et al. (2022), Sharma and Chang (2017)
*SHH*	Hedgehog signalling	Downstream gene of the SHF transcriptional cascade.	Mouse	–	Wang et al. (2016), Qyang et al. (2007), Ren et al. (2021)
*SHOX2*	Homeobox TF	TBX5-activated; maintains SAN signature; co-enriched in human SAN-related gene set.	Mouse + human	–	Park and Fishman (2026), Kapoor et al. (2013)
*SIX2*	Homeobox TF	Direct transcriptional target of TBX1 in the cardiopharyngeal mesoderm.	Mouse	–	Nomaru et al. (2021)
*SMAD1*	BMP signalling effector	Activated downstream of BMP4 to mediate transcriptional responses during cardiac mesoderm induction.	Mouse	–	Tsaytler et al. (2023)
*TBX1*	T-box TF	Acts in the cardiopharyngeal mesoderm; primes a bipotent SHF–branchiomeric population; regulates proliferation/differentiation of SHF precursors at the arterial pole; ChIP-seq targets include *ISL1, FOXC2, SIX2*.	Mouse + human	22q11.2 deletion syndrome conotruncal/ OFT malformations	Baldini et al. (2017), Nomaru et al. (2021), De Bono et al. (2023)
*TBX2*	T-box repressor	Confined to non-chamber myocardium of the AVC; restricted by TBX20; inhibited by Notch-activated HEY1/HEY2 in chamber myocardium; repressed by HEY2 in ventricular myocardium.	Mouse	–	Seya et al. (2021), Chen et al. (2021), Li et al. (2021), Park and Fishman (2026)
*TBX3*	T-box repressor	Essential for differentiation and maintenance of the CCS; activated by TBX5 to maintain SAN signature.	Mouse + human	Ulnar-mammary syndrome	Park and Fishman (2026), Mohan et al. (2018), Kapoor et al. (2013)
*TBX5*	T-box TF	FHF marker; cooperates genome-wide with GATA4 for chamber specification; drives the atrial programme (*NPPA, GJA5, BMP10*);	Mouse + human	Holt–Oram syndrome	Miyamoto et al. (2021a), Canac et al. (2022), Ang et al. (2016), Gonzalez-Teran et al. (2022), Wang et al. (2013), Sweat et al. (2023), Park and Fishman (2026)
*TBX18*	T-box repressor	Represses *GJA1* (CX43) and can induce SAN-like features in ventricular cardiomyocytes; marks a pro-epicardial subpopulation directed to vascular smooth muscle.	Mouse + human	–	Park and Fishman (2026), Kapoor et al. (2013), Feulner et al. (2022), Sharma and Chang (2017)
*TBX20*	T-box TF	MESP1 target; inhibits *TBX2* to confine it to non-chamber myocardium and downregulates *TBX5*; cooperates with TBX5/GATA4/NKX2-5 in fast-conduction gene programmes.	Mouse	–	Bondue and Blanpain (2010), Bondue et al. (2008), Wang et al. (2016), Qyang et al. (2007), Ren et al. (2021), Chen et al. (2021), Park and Fishman (2026)
*WNT3*	Wnt signalling ligand	Mutually antagonistic feedback with CERL2 at the LRO.	Mouse	–	Little and Norris (2021)
*WT1, GATA5, TCF21*	Pro-epicardial transcription factors	Mark pro-epicardial subpopulations directed toward vascular smooth muscle cell differentiation (together with TBX18).	Mouse (lineage tracing)	–	Feulner et al. (2022), Sharma and Chang (2017)
*YAP, TAZ*	Hippo pathway effectors	Regulators of EMT enabling epicardium-derived cells to invade the myocardium and support coronary vessel maturation.	Mouse	–	Feulner et al. (2022), Sharma and Chang (2017)
*ZIC3*	Zinc-finger TF	Critical for LRO structure during gastrulation; established factor in human laterality.	Mouse + human	X-linked heterotaxy (∼75% of families; ∼3% of sporadic males)	Sempou and Khokha (2019), Wells et al. (2022)

Gene symbols are given in human HGNC nomenclature; in the body text they follow the convention of the species of origin. LRO, left-right organizer; FHF, first heart field; SHF, second heart field; LV, left ventricle; ECM, extracellular matrix; LPM, lateral plate mesoderm; CCS, cardiac conduction system; SAN, sinoatrial node; TF, transcription factor.

## Cardiac embryology

**FIGURE 1:**
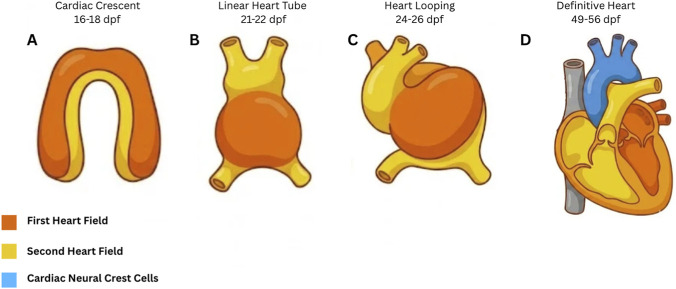
Lineage contributions to the developing heart across four morphogenetic stages. Schematic representation of the cellular origins of the cardiac chambers and great vessels, colour-coded by progenitor population: first heart field (FHF, orange), second heart field (SHF, yellow), and cardiac neural crest cells (CNCCs, blue); grey denotes non-myocardial, non-CNCC tissue. **(A)** Cardiac crescent (16–18 dpf): the FHF (orange) forms the crescent, with the SHF (yellow) positioned medially. **(B)** Linear heart tube (21–22 dpf): FHF-derived cells (orange) constitute the primitive tube, with SHF-derived cells (yellow) added at the arterial and venous poles. **(C)** Heart looping (24–26 dpf): the tube loops rightward, with FHF (orange) and SHF (yellow) contributions becoming regionally distinct. **(D)** Definitive heart (49–56 dpf): mature four-chambered heart showing FHF (orange), SHF (yellow), and CNCC (blue) derivatives, together with non-myocardial, non-CNCC tissue (grey). dpf, days post-fertilisation.

One of the key processes of embryogenesis is the determination of anteroposterior, dorsoventral and left-right embryonic axes. Another crucial and early aspect is the specification of the germ layers: endoderm, mesoderm, and ectoderm, as well as the subsequent patterning of cell fates along the axes (Solnica-Krezel and Sepich, 2012). A simplified diagram of the cardiogenesis is shown in Figure 1.

The heart is the first functional organ to develop post-conception, but this process is somewhat complex and involves the interaction and assembly of various cell types (Miyamoto et al., 2021a) with molecular signalling and cell-to-cell interactions playing a significant role in cardiac formation. It encompasses cells from three central precursor regions: the cardiac mesoderm, the proepicardium, and the cardiac neural crest (Li et al., 2024).

Myocardial progenitor cells arise from the mesoderm, migrating anteriorly and laterally to form the anterior lateral plate mesoderm, commonly referred to as *the cardiac crescent* (Dabbagh et al., 2017; Galdos and Wu, 2019). In this crescent, two molecularly distinct populations of cardiac progenitor cells are specified and named based on the timing of their contribution as *first and second heart fields* (FHF and SHF, respectively) (Miyamoto et al., 2021a). As the FHF cells form the heart crescent *per se*, the SHF lies more medial and dorsal (Galdos and Wu, 2019; Galdos et al., 2017), and it is in close contact with the underlying endoderm, which is thought to signal the maintenance and deployment of SHF progenitors (Shewale and Dubois, 2021). Both lineages develop in proximity and without clear boundaries, but with distinct properties and biomolecular markers (Miyamoto et al., 2021a).

The FHF flaps fold towards the embryo’s ventral midline and fuse cranially-caudally in a zipper-like manner: the lateral parts are brought together, forming the ventral face, while the inner curvature forms the dorsal side, which is contiguous with the foregut endoderm through the dorsal mesocardium, resulting in the formation of a myocardial mantle that encloses the two primitive endocardial primordia (Abu-Issa, 2014; Laura et al., 2020; Sylva et al., 2014).

Eventually, the endocardial primordia fuse, and the dorsal part of the myocardial hemi-tube detaches from the posterior pericardial wall, forming a three-layered structure that contains the endocardic and myocardic tubes separated from each other by a layer of the acellular matrix of hygroscopic molecules known as *cardiac jelly* (Christoffels and Jensen, 2020; Harris and Black, 2010).

During their migration, the FHF cells differentiate in cardiomyocytes and stop proliferating upon forming the heart tube (Laura et al., 2020; Christoffels and Jensen, 2020; Günthel et al., 2018), which increases four to five-fold during the following stages of development, mainly by the addition of the rapidly proliferating mesodermal SHF precursor cells (Christoffels and Jensen, 2020; Günthel et al., 2018).

The anatomical contribution of each embryologic segment to the mature heart remains controversial (Laura et al., 2020). However, it is usually assumed that the FHF contributes primarily to the left ventricle (LV) with some involvement in the development of the interventricular septum and the atrioventricular canal (Laura et al., 2020; Villavicencio-Guzmán et al., 2023), whereas the SHF, with its remarkable pluripotency, can give rise to most of the right ventricle (RV), the conotruncal region, and endothelial and myocardial components of the outflow tract (OFT) (Miyamoto et al., 2021a; Dabbagh et al., 2017; Shewale and Dubois, 2021). The significant involvement of precursor cells beyond the initial heart tube may explain the frequent co-occurrence of CHDs and extracardiac anomalies (Calcagni et al., 2021).

During their addition to the heart tube, the SHF-derived cells also temporally cease proliferation (Günthel et al., 2018). The heart tube increases four- to fivefold during the next stages while undergoing bending and rotation to form a C-looped and, later, an S-shaped tube. Successful looping is essential for properly aligning the future cardiac chambers and inflow and outflow tracts (Christoffels and Jensen, 2020; Ebrahimi et al., 2022). When looping is complete, the heart assumes its basic configuration, and then extensive morphological remodelling follows, including trabeculation, chamber septation, cushion, and valve formation (Ebrahimi et al., 2022).

Chamber myocardium arises from both heart fields; most likely induced within the primary myocardium in response to positional cues. While the non-chamber myocardium can undergo spontaneous depolarisation, chamber cells exhibit slower electrical conduction and are better adapted for mechanical work. The specification of chamber and non-chamber myocardium in the forming heart is a crucial early lineage differentiation upon which much of the subsequent development is based (Stennard and Harvey, 2005).

The individual cardiac chambers and the epicardium are completely formed around 30 days post-fertilization, followed by the differentiation of the OFT into the aorta and pulmonary artery and compaction of the ventricular myocardium. Most cardiac compartments are complete by the end of the first trimester (Asp et al., 2019).

Overall, the heart is essentially a mesodermal derivative, although some regions, such as the outflow tract, also contribute neural crest cells derived from the ectoderm (Sylva et al., 2014). The neural crest cells arise from the dorsal neural tube, an ectodermal structure, and are a highly migratory, pluripotent cell population. Cardiac Neural Crest Cells (CNCCs) are a subpopulation that contributes to the posterior aortic arteries' perivascular walls, which are indispensable for the formation and septation of the proximal OFT (Stefanovic et al., 2021; Keyte et al., 2014).

Hence, epithelial-to-mesenchymal transition (EMT) is crucial from the beginning of cardiogenesis, as all cells arise from one or more EMTs during gastrulation and through cardiac development. EndoMT generates valve progenitor cells and is necessary for complete cardiac septation (Von Gise and Pu, 2012).

During development, the embryonic heart depends critically on both its electrical and mechanical functions. Processes such as heart tube remodelling, looping, chamber expansion, and alignment generate localised variations in shear stress, pressure, and tension. These mechanical cues are detected by mechanosensors, which play a pivotal role in activating specific signalling pathways within endocardial and underlying myocardial cells (Christoffels and Jensen, 2020).

### Transcriptional and signalling pathways in early cardiogenesis

The complex process of heart formation demands precise temporal, spatial, and cell-type-specific regulation mediated by transcription factors (TFs) and their interacting regulatory networks (Canac et al., 2022). Spatial and temporal specificity can be achieved by forming multiprotein complexes that contain a subset of these factors, along with other induced or ubiquitous components (Gallagher et al., 2012). Such TFs form complexes with other tissue-enriched proteins to orchestrate specific developmental programs; variants affecting these interactions may disrupt genome-wide patterns of transcriptional activation and repression, thereby impairing cardiac lineage specification (Ang et al., 2016; Gonzalez-Teran et al., 2022).

Although complete knowledge of the cardiac development network is still lacking, a great deal of data has been released in the last decade due to new technologies, such as single-cell RNA sequencing (scRNA-seq), spatial transcriptomics and *in situ* sequencing (ISS), organoids and human induced pluripotent stem cells (hiPSCs). Around 216 TFs were found to be differentially expressed during the time course of cardiogenesis, of which 69% were linked to cardiac phenotypes (Canac et al., 2022).

**FIGURE 2:**
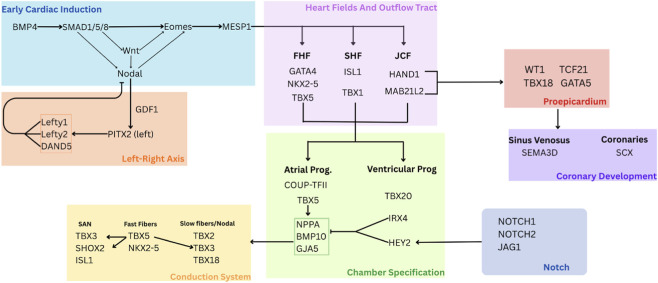
Map of cardiogenesis: a developmental hierarchy of signalling pathways, transcription-factor networks, and progenitor populations underlying normal heart formation and disrupted in congenital heart disease**.** Solid arrows denote activation; T-bars denote inhibition. FHF: First Heart Field; SHF: Second Heart Field; CNCCs: Cardiac Neural Crest Cells; SAN: sinoatrial node.

The main genes discussed in this paper are represented in Figure 2.

### Early transcriptional hierarchy

In mammals, mesodermal lineage cells are derived from pluripotent epiblast cells that undergo EMT in the primitive streak (PS). Cardiac mesoderm progenitors are derived from the proximal PS, and several inducers are involved, in particular members of the TGF-β family and canonical Wnt signalling, each activating their distinct transcriptional programs (Tsaytler et al., 2023).

Cardiac mesoderm induction depends in part on TGF-β superfamily signalling, particularly *BMP4*, which activates SMAD1/5-mediated transcriptional responses. Together with Wnt and Nodal signalling, this feedforward network promotes the emergence of early cardiac mesoderm and precedes Eomes activation (Tsaytler et al., 2023).

The earliest known committed cardiac precursors express the T-box TF *Eomesodermin* (*Eomes*), which specifically contributes to the cardiovascular system by activating *MESP1*, enabling cardiac progenitors to emerge from the primitive streak, migrate anteriorly, and establish the cellular substrate from which the heart fields will arise (Bondue and Blanpain, 2010; Bruneau, 2013).

In fact, the *MESP1* ^*+*^ lineage is much broader, encompassing almost all the trunk mesenchyme and the vasculature (Bruneau, 2013), and it massively promotes cardiovascular differentiation and EMT during embryonic development. MESP1 resides at the apex of the transcriptional hierarchy, orchestrating the emergence, migration, and early segregation of cardiac progenitors that will subsequently assemble the primitive heart tube. Mesp1 rapidly promotes the expression of most cardiovascular TFs–such as *Hand2, Myocd, Nkx2-5, Gata4, Mef2c, Tbx20, Foxh1, Foxc1* and *Foxc2* – by binding directly to their promoter regions (Bondue and Blanpain, 2010; Bondue et al., 2008).

In the absence of *MESP1*, *MESP2*, the closest homolog located on the same chromosome and separated by only 23 kb, is massively upregulated in cardiac mesoderm and may partially compensate for the *MESP1* loss (Bondue and Blanpain, 2010).

### Heart fields regulatory networks

Both FHF and SHF are specified around the onset of *MESP1* expression. FHF progenitors are less proliferative and primarily differentiate into cardiomyocytes, expressing TFs such as *Tbx5, Gata4, Nkx2-5,* and *Hand1* (Miyamoto et al., 2021a). Crucially, *Tbx5* and *Gata4* cooperate genome-wide to regulate lineage-specific transcriptional programs required for chamber specification, particularly those associated with left ventricular and septal development (Gonzalez-Teran et al., 2022). These interactions are also temporally ordered: GATA4, NKX2-5, and TBX5 act sequentially during early cardiac differentiation, with *Gata4* expression preceding *Nkx2-5* and *Tbx5* (Canac et al., 2022).

Mechanistic studies show that heterozygosity for a *Gata4* missense mutation disrupts this cooperative interaction, impairing GATA4/TBX5-mediated repression of non-cardiac genes. As a result, chromatin becomes more accessible at regions that promote endothelial and endocardial gene expression (Ang et al., 2016; Gonzalez-Teran et al., 2022). GATA4 regulates transcriptional programs in cardiomyocytes, endothelial cells, and fibroblasts. Accordingly, GATA4 regulates cardiomyocyte proliferation and survival, maintains vascular integrity and remodelling, and modulates extracellular matrix production (Heuvelmans et al., 2025).

Clinically, heterozygous pathogenic variants in *TBX5* cause septation defects and other forms of CHD in the setting of Holt-Oram syndrome, which is also associated with upper limb malformations (Gonzalez-Teran et al., 2022).

Likewise, the broad and persistent expression of *GATA4* throughout cardiogenesis supports its central role in heart development and function, and pathogenic variants in this gene have been associated with a wide spectrum of CHD phenotypes, including septal, valvular, and outflow tract malformations (Heuvelmans et al., 2025).

The hyperpolarisation-activated nucleotide-gated cation channels (HCN 1-4) govern cell membrane excitability in neurons, smooth muscles and heart. HCN4 is the predominant cation channel in cardiac sinoatrial (SAN) pacemaker cells and is expressed in the first differentiating cells of the cardiac crescent and transiently throughout the early heart tube, marking the FHF in a complementary expression domain to *Isl1* (Liang et al., 2013; Wu et al., 2025).

In contrast to FHF, SHF progenitors are highly proliferative, delayed in their differentiation, and unequivocally marked by *Isl1* expression. During mammalian cardiogenesis, *Isl1* works in concert with the Brg1/Baf60c chromatin remodelling complex to shape the epigenetic landscape and regulate the expression of target genes in cardiac progenitor cells. *Isl1* activity supports SHF progenitor maintenance and deployment, thereby promoting progressive cell addition to the arterial pole and contributing to right ventricular and outflow tract formation (Wang et al., 2016).

The SHF regulatory network contains three main axes: 1) cooperates with forkhead TF FOXH1 to set in motion a cascade of transcriptional events involving downstream genes such as *Mef2c*, *Myocd*, *Nkx2-5*, *Shh,* alongside key regulators of cardiac development like *Gata4* and *Tbx20*; 2) regulates essential signalling molecules such as FGF10 and BMP4, critical for the SHF development; and 3) promotes expression of genes involved in cardiomyocyte structure and contraction: *Ttn, Ryr2, Myl3* (Wang et al., 2016; Qyang et al., 2007; Ren et al., 2021).

Spatially and temporally, as SHF progenitors migrate to join the arterial and venous poles, *ISL1* is actively expressed in most cells destined to form the right ventricle and the outflow tract (OFT), as well as partially in the atria. As cardiac cells differentiate, *ISL1* expression progressively declines and becomes restricted to a subdomain of the right atrium, linking its later expression pattern to the emergence of the pacemaker region and the early specialisation of the cardiac conduction system. (Wang et al., 2016; Ren et al., 2021).

Closely intertwined with the *ISL1* ^+^ SHF population is the Cardiopharyngeal Mesoderm (CPM). Within this niche, *TBX1* acts as a critical transcriptional activator. Contrary to expectations based on the phenotype of 22q11.2 deletion syndrome, *Tbx1* in mice is expressed in the head mesoderm rather than in neural crest cells or the cardiac mesoderm itself. Its primary role is to regulate the proliferation and differentiation of SHF precursors destined to populate the arterial pole of the heart, thereby contributing to outflow tract elongation and alignment (Baldini et al., 2017; Nomaru et al., 2021; De Bono et al., 2023).

Clonal analysis and lineage-tracing studies reveal that *Tbx1* primes a bipotent, multilineage population within the CPM, capable of generating both SHF-derived cardiac cells and branchiomeric skeletal muscles of the craniofacial and neck regions. Thus, *Tbx1* is required for these multipotent precursor cells to express critical genes for cardiac development while preventing ectopic transcription of non-mesodermal genes (Nomaru et al., 2021).

Mechanistically, this regulation is direct: ChIP-seq analysis suggests that *ISL1, FOXC2*, and *SIX2* are direct transcriptional targets of TBX1. This hierarchical relationship emphasises the fragility of the OFT development and helps explain why *TBX1* deficiency is strongly associated with conotruncal defects; indeed, given the sophisticated regulation of TBX1 during embryogenesis, loss-of-function variants or CNVs are associated with a wide range of OFT malformations (Nomaru et al., 2021).

### Integrating cardiogenesis: the Notch pathway

Notch signalling contributes to cardiogenesis across multiple stages, from early cardiac mesoderm patterning and heart field specification to later morphogenetic events, including endocardial cushion EMT, valve formation, outflow tract development, and ventricular trabecular compaction. This broad temporal activity highlights its role as a regulatory bridge between progenitor allocation and structural remodelling of the forming heart (Ye et al., 2023). It has been suggested that Notch may influence multiple processes of early development by regulating Wnt signalling, which plays a key inductive role in primitive streak and cardiac mesoderm formation (Miyamoto et al., 2021b).

Studies with hiPSCs have shown that *NOTCH1* disruption promotes epicardial and SHF cell fate determination but inhibits the differentiation of FHF progenitors from cardiac mesoderm (Ye et al., 2023). Reduced *Notch1* activity in the SHF leads to abnormal migration of CNCCs and defective EMT within the OFT endocardial cushions (High et al., 2009). Noncanonical Notch signalling suppresses SHF formation in the precardiac mesoderm of murine models, suggesting a biphasic role in cardiac progenitor cells: inhibiting SHF in early development and promoting cardiomyocyte proliferation in later stages (Miyamoto et al., 2021b).

Given its central regulatory role, abnormal Notch signalling or loss-of-function in pathway components can lead to CHD phenotypes, including ventricular septal defects and semilunar valve malformations. Beyond NOTCH1, pathogenic variants in the Notch ligand *JAG1* — and, less commonly, in *NOTCH2* — underlie Alagille syndrome in humans, an autosomal-dominant multisystem disorder characterised by chronic cholestasis due to intrahepatic bile duct paucity, characteristic facies, butterfly vertebrae, and a broad spectrum of cardiovascular anomalies (Liu et al., 2026; Luxán et al., 2016).

### Emerging progenitor populations

Beyond the classical FHF and SHF binary, recent transcriptomic analyses have identified a distinct subpopulation of primordial cells, the Juxtacardiac Field (JCF). Located at the confluence of the splanchnic and extraembryonic mesoderm, rostral to the cardiac crescent, the JCF also derives from the *Mesp1* ^+^ lineage and is characterised by *Hand1* expression yet notably lacks robust *Nkx2-5* expression (Sendra et al., 2022; Tyser et al., 2021). Interestingly, during early gastrulation, *Hand1* marks a progenitor population that not only produces cardiomyocytes destined for the atrioventricular canal and the dorsolateral region of the left ventricle, but also yields mesodermal cells for the pericardium, epicardium, and extra-embryonic tissues. This broad developmental potential suggests that the FHF may comprise two distinct populations (Zhang et al., 2021).

A defining transcriptomic signature of the JCF is *Mab21l2*, a highly conserved gene involved in cell proliferation. In murine models, as JCF cells migrate toward the cardiac crescent to contribute to the heart tube—particularly to left ventricular progenitor territories—*Mab21l2* is downregulated while *Nkx2-5* expression is activated (Sendra et al., 2022; Tyser et al., 2021).

Crucially, following heart tube formation, the remaining *Mab21l2* ^+^ cells become restricted to the dorsal mesoderm, acting as a major source for the pro-epicardium and subsequently expressing markers such As *Bnc2*, *Wt1*, and I*tga8*. However, it remains a subject of ongoing debate whether the JCF constitutes a cluster of distinct unipotent progenitors—destined separately for cardiomyocytes and the pro-epicardium—or if it harbours bipotent cells capable of generating both lineages. The contribution of JCF-derived cells to both cardiomyocyte and proepicardial lineages suggests that this population may participate not only in early ventricular patterning but also in the later establishment of the epicardial covering that supports coronary vessel development (Tyser et al., 2021; Snabel et al., 2025).

### Crossroads: the left-right axis

Establishing the left-right axis is a multi-step process, and the heart is the first organ to break morphological symmetry. However, the molecular events underlying this asymmetry begin even before heart tube formation, when directional cues are sensed and translated into differential gene expression around a transient midline organiser known as the Left-Right Organiser (LRO). Central to all vertebrates, the LRO consists of a ciliated epithelium in and adjacent to the primitive or central node of the planar embryo (Djenoune et al., 2022; Dykes, 2014).

The TF *ZIC3* is critical for the LRO structure due to its activity in the migrating mesoderm during gastrulation and is an established factor in human laterality. Correct patterning of LRO progenitor cells in the gastrula also requires canonical Wnt signalling (Sempou and Khokha, 2019).

The cilia in the LRO have a critical role in establishing the left-right (LR) asymmetry by generating leftward flow and defining proper placement and patterning of the internal organs, including heart looping (Djenoune et al., 2022; Desgrange et al., 2018). Proper leftward fluid flow depends on planar cell polarity, which is regulated by non-canonical Wnt signalling and plays a critical role in cell shape and convergent extension. Defects in this pathway disrupt LR patterning and can also cause neural tube defects in multiple model organisms (Wells et al., 2022).

The precise mechanism by which this movement is perceived is uncertain and likely involves both chemo- and mechanosensory mechanisms. However, it is clear that the flow regulates divergent signalling: differential gene expression is first observed in the crown cells at the periphery of the node. Nodal, a secreted morphogen of the TGF-β/BMP superfamily, is expressed strongly on the left side, while expression of Nodal inhibitor *Cerl2* shows the opposite distribution (Djenoune et al., 2022; Dykes, 2014). Mutually antagonistic feedback exists between *Wnt3* and *Cerl2* (Little and Norris, 2021).

Once asymmetrically expressed, Nodal reinforces its own expression, thereby establishing the LR programme. Transcriptomic analysis confirms the biased targets of Nodal signalling, which modulate proliferation, differentiation, and extracellular matrix composition, providing mechanisms by which Nodal may generate and amplify left-right asymmetries (Desgrange et al., 2018).

Nodal expression propagates from the LRO into the left-lateral plate mesoderm (LPM) by forming a heterodimer with GDF1 (Wells et al., 2022). This pathway utilises core Smad-dependent signalling components and cooperates specifically with *Foxh1*. Amplification of the Nodal signalling culminates in the expression of the TF PITX2 (Paired-like homeodomain 2), the primary downstream effector of left-sidedness. *Pitx2* drives asymmetric morphogenesis at the arterial and venous poles, ensuring the correct looping of the linear heart tube (Desgrange et al., 2018; Kato, 2011).

Nodal antagonists act in distinct embryonic domains to spatially restrict the left-sided programme. Within the LRO, *Dand5,* a BMP antagonist, is a well-characterised LR cue that is asymmetrically expressed in the right side, where it competes with Nodal and prevents inappropriate activation of left-sided signalling (Sempou and Khokha, 2019).

*Lefty1* is expressed at the embryonic midline in response to Nodal. Loss of *Lefty1* function leads to bilateral Nodal expression followed by ectopic induction on the right side, indicating that it acts as a midline barrier that prevents Nodal signalling from spreading to the right side of the embryo (Little and Norris, 2021).

Another Nodal antagonist, *Lefty2*, is induced by *Pitx2* and functions as a Nodal feedback inhibitor in the LPM, restricting the self-enhancing loop of Nodal signalling both spatially and temporally, thereby limiting Nodal effects to a short time window (Sempou and Khokha, 2019). While *Nodal* and *Lefty2* are expressed transiently, *Pitx2* remains present during organogenesis. Loss of *Pitx2* leads to right pulmonary and atrial isomerism and randomisation of gut situs (Desgrange et al., 2018; Little and Norris, 2021).

Heterotaxy spectrum disorders are characterised by an abnormal arrangement of the thoracoabdominal organs caused by disruption of left-right patterning. These defects may result in atrial isomerism, abnormal cardiac looping, and complex congenital heart defects, particularly those affecting atrioventricular connections and outflow tract alignment.

Monogenic causes of heterotaxy can be associated with decreased penetrance and variable expressivity. Mutations affecting the Nodal/TGF-β signalling are often autosomal dominant. However, autosomal recessive variants in the Nodal co-ligand *GDF1* and in the Nodal antagonist *DAND5* have also been reported. Pathogenic variants in the *ZIC3* gene are identified in 75% of families with X-linked heterotaxy and 3% of males with no family history (Wells et al., 2022).

### End of the road: chamber specification, conduction and coronary development

#### Chamber morphogenesis and atrial-ventricular identity

Once the linear heart tube has undergone looping, the simple conduit must transform into a complex, four-chambered pump capable of sustaining separate pulmonary and systemic circulations. This morphological diversification occurs through a ballooning mechanism, in which distinct atrial and ventricular chambers expand rapidly from the outer curvature of the linear tube. Crucially, this expansion relies on the precise spatial restriction of gene expression: while the outer curvature differentiates into rapidly proliferating working myocardium, the inner curvature and the atrioventricular canal retain a primitive, slowly proliferating phenotype that eventually gives rise to the septa and components of the conduction system (Yao et al., 2024).

At the venous pole, atrial specification is safeguarded by the nuclear receptor Nr2f2 (COUP-TFII), widely recognised as the most prominent determinant of atrial identity in vertebrates (Martin and Waxman, 2021). Genome-wide analyses reveal that NR2F2 binds to loci within 50 kb of genes associated with cardiac morphogenesis, directly modulating the expression of atrioventricular identity genes such as *Tbx5, Hey2*, and *Irx4* (Wang et al., 2013). Concomitantly, TBX5 acts as a potent driver of the atrial program, promoting the expression of specific markers including *Nppa* (ANF), *Gja5* (Cx40), and *Bmp10*. Recent mechanistic insights demonstrate that TBX5 interacts directly with chromatin remodelling subunits CHD4 and SMARCD3, a cooperation essential for gaining access to these target genes and establishing the specific morphology and electrophysiological profile of the atrial chambers (Sweat et al., 2023).

Conversely, in the expanding ventricular chambers, the myocardium acquires its specific identity through a robust program of activation and repression. The HEY2 transcription factor is prominently expressed in the ventricular myocardium and endocardium and acts as a crucial determinant of ventricular morphology. HEY2 and other Hey proteins directly repress atrial-specific genes (such as *Nppa* and *Gja5*), *Gata4/6*, and *Tbx2*, thereby preventing ectopic atrial programs and driving appropriate myocardial maturation (Seya et al., 2021). Furthermore, *Tbx20* plays an essential role in chamber specification by inhibiting *Tbx2*—confining its expression to the non-chamber myocardium of the atrioventricular canal—and downregulating the canonical atrial gene *Tbx5* (Chen et al., 2021). Loss of myocardial *Hey2* function disrupts the rightward shift of the atrioventricular cushion and can lead to severe cardiac phenotypes, including tricuspid atresia (Okumura et al., 2024).

The Iroquois homeobox (IRX) family of TFs is implicated from the earliest stages of cardiac progenitor specification through the end of differentiation. Although most IRX factors are expressed from early progenitor stages onward, their developmental relevance becomes particularly evident during chamber patterning, where they help establish regional ventricular identity and electrophysiological properties (Kim et al., 2012).

In hiPSC models, *IRX3, IRX4*, and *IRX5* occupy distinct temporal positions during cardiac differentiation, supporting partially overlapping roles in ventricular specification and electrical maturation, whereas *IRX6* was undetectable and *IRX1-2* did not vary over time. Based on their expression profiles, *IRX3* and *IRX5* were detected from the earliest cardiac-specific gene cluster until the end of cardiac hiPSC differentiation, while *IRX4* was detected in one of the latest clusters (Canac et al., 2022). IRX4 is required for the establishment of some components of the ventricular gene expression program by promoting *Hand1* expression while suppressing *Nppa* and *Acta1* (Canac et al., 2022; Nelson et al., 2016).

#### Notch pathway in septation, valve morphogenesis and ventricular maturation

Beyond chamber-specific transcription factors, broader signalling pathways continue to shape septation, valve morphogenesis, and ventricular maturation. Notch1 is essential for human ventricular differentiation and septation by balancing cell fate determination of the early cardiac mesoderm toward epicardial, FHF, and SHF lineages while restricting atrial cardiomyocyte generation (Li et al., 2021).

A crucial morphogenetic role of Notch signalling is the regulation of endocardial cushion EMT, a process required for atrioventricular septation and valve formation that helps transform the primitive heart tube into a septated, valved organ. Notch-activated *Hey1* and *Hey2* can inhibit *Bmp2* and the downstream *Tbx2* in the heart chambers, thereby limiting their expression specifically in the AVC. In the OFT, Notch signalling also influences CNCC–endocardial interactions and cushion remodelling, thereby contributing to arterial pole morphogenesis and semilunar valve formation (Li et al., 2021).

In hiPSC models, disruption of *NOTCH1* or downstream loss of *HEY2* activity promotes an atrial-like phenotype at the expense of ventricular cardiomyocyte differentiation through upregulation of COUP-TFII. Moreover, Notch signalling exhibits stage-specific effects on ventricular development, including trabeculation and compaction. These hiPSC models may help explain the role of *NOTCH1* variants in a spectrum of left-sided malformations, including Hypoplastic Left Heart Syndrome (Ye et al., 2023).

#### Chamber-specific electrical patterning and conduction system emergence

Cardiac Conduction System (CCS) development emerges from progressive restriction of chamber myocardial potential toward distinct fast- and slow-conducting lineages, linking morphogenesis to electrical specialisation. Its specification, expansion, and patterning are governed by an interconnected hierarchy of transcriptional networks.

TBX factors, particularly *TBX5* and *TBX20*, cooperate with *GATA4* and *NKX2-5* to promote the electrical and chamber-specific gene programs required for fast-conducting myocardium, including genes such as *GJA5* and *SCN5A*. Conversely, *TBX2, TBX3,* and *TBX18* are repressors that maintain a slow-conduction, nodal-specific gene profile (Park and Fishman, 2026).

*TBX5* is essential for both fast- and slow-conducting cardiac tissues, and its effects vary depending on the cofactors present in each developmental context. *TBX5* is also crucial for maintaining the SAN signature, as it activates the expression of *TBX3* and *SHOX2*. This regulatory role helps explain why patients with Holt–Oram syndrome frequently display varying degrees of CCS dysfunction (Park and Fishman, 2026).

*IRX3* and *IRX5* participate in a time-dependent transcriptional cascade involving *GATA4, NKX2-5*, and *TBX5*, culminating in *SCN5A* expression. Because *SCN5A* encodes the alpha subunit of the major cardiac sodium channel, this regulatory axis links ventricular patterning to the acquisition of chamber-specific electrical properties during cardiomyocyte differentiation (Canac et al., 2022).

In humans, *HCN4, HCN1* and *HCN2* are expressed throughout the atria, with *HCN1* almost exclusively expressed in SAN, while both *HCN2* and *HCN4* are present in both SAN pacemaker cells and surrounding right atrial myocytes (Hennis et al., 2021). In murine models, the earliest *Hcn4*-expressing cells contribute to a few cells in the SAN tail, but later *Hcn4+* cells from the posterior SHF form most of the node. *Hcn4* is also expressed in the early precursors of Purkinje and His fibres but is later downregulated and remains only in the central components of the CCS (Liang et al., 2013).

The ventricular conduction system is a unique component of the CCS in which fast-conduction programmes, regulated in part by *TBX5* and *NKX2-5*, coexist with restrictive mechanisms involving *TBX3*, thereby contributing to a distinct Purkinje cell identity (Park and Fishman, 2026). *TBX3* is essential for differentiation and maintenance of the conduction system. During development, the CCS is established through progressive fate restriction of a *TBX3* ^*+*^ cell population that forms a specialised network distinct from the early GJA5^+^ chamber myocardium (Mohan et al., 2018).

*TBX3* is expressed throughout the developing and mature CCS, except for Purkinje fibre network, and is associated in human hearts with enrichment of SAN-related genes such as *ISL1, SHOX2,* and *BMP4*. TBX18 represses *GJA1* (CX43) expression and can induce SAN-like features in ventricular cardiomyocytes, a feature that has been considered for engineering biological pacemakers (Park and Fishman, 2026; Kapoor et al., 2013).

#### Coronary vessel development

Finally, the establishment of the coronary vasculature ensures the perfusion of the progressively thickened myocardium. This process depends on coordinated interactions among the epicardium, endocardium, and multiple progenitor populations. The proepicardium gives rise to the epicardium, which subsequently contributes cells to the mural components of the coronary vessels, particularly smooth muscle cells and perivascular fibroblasts. In contrast, the origin of the coronary endothelium is more heterogeneous and remains an area of active investigation, with contributions proposed from distinct vascular and endocardial-associated sources (Borasch et al., 2020; Feulner et al., 2022).

In murine models, lineage tracing reveals a sophisticated compartmentalisation of pro-epicardial cells with distinct developmental fates: *Sema3d* ^*+*^ and *Scx* ^*+*^ populations contribute, respectively, to the sinus venosus and the coronary vascular lineage, whereas *Wt1* ^*+*^ *, Gata5* ^*+*^ *, Tcf21* ^*+*^ *,* and *Tbx18* ^*+*^ subpopulations are directed towards vascular smooth muscle cell differentiation (Feulner et al., 2022; Sharma and Chang, 2017).

Mechanistically, coronary vascular development also depends on EMT, which enables epicardium-derived cells to invade the myocardium and support vessel maturation. Hippo pathway effectors such as YAP and TAZ are important regulators of this process. In parallel, endocardial signalling plays a central role in coordinating cardiac morphogenesis and vascular development by modulating myocardial growth, trabeculation, and communication between intracardiac tissues (Feulner et al., 2022; Sharma and Chang, 2017).

## Conclusions and future perspectives

Heart formation is a highly coordinated developmental process in which transcription factors, signalling pathways, epigenetic regulators, and tissue-specific cellular interactions act in a precise temporal and spatial manner. Although advances in cytogenetics, single-cell approaches, and computational biology have greatly expanded our understanding of cardiogenesis, the precise mechanisms underlying tissue-type morphogenesis in normal or defective human hearts remain to be elucidated. By integrating embryological events with their underlying regulatory networks, this review highlights how disruption of specific developmental modules may give rise to congenital heart disease. A more comprehensive map of cardiogenesis will be essential not only for refining diagnosis and prognosis but also for guiding future strategies in disease modelling, risk stratification, and regenerative medicine.
